# Visioning services for children affected by HIV and AIDS through a family lens

**DOI:** 10.1186/1758-2652-13-S2-I1

**Published:** 2010-06-23

**Authors:** Linda Richter, Chris Beyrer, Susan Kippax, Shirin Heidari

**Affiliations:** 1 Child Youth Family & Social Development (CYFSD), Human Sciences Research Council, Durban, South Africa; 2 Department of Epidemiology, Bloomberg School of Public Health, The Johns Hopkins University, Baltimore, USA; 3 Social Policy Research Centre, University of New South Wales, Sydney, Australia; 4 International AIDS Society, Geneva, Switzerland

## Abstract

The HIV epidemic continues to place a great burden on children, from loss of parents and income to severe disruptions of their homes and families. Underpinned by the understanding that a healthy family constitutes the foundation for a child's wellbeing, the importance of family-centred care and services for children is increasingly recognized. It is not enough to merely provide antiretrovirals: it is of pivotal importance that treatment and care for children are integrated into the broader context of family-support schemes. However, despite growing evidence of the benefits of family-centred services, reforms in favour of family oriented HIV interventions have been slow to emerge. Treatment, prevention and care interventions often target individuals, and not families and communities.

For the first time, this supplement to the *Journal of the International AIDS Society *brings together in one place the rationale for family-centred services for children affected by HIV and AIDS and some of the available evidence for the effectiveness of doing so. We hope this constitutes a beginning of what could be a groundswell of interest in family-centred services for children affected by HIV and AIDS.

## 

Children have borne a significant burden throughout the course of the HIV epidemic. Countless children have been infected with the virus as a result of failure in rollout of interventions that prevent vertical transmission. Globally, two million children under the age of 15 are estimated to be living with HIV [1].

In 2008, the coverage of prevention of mother to child transmission (PMTCT) programmes was around 45% in low- and middle-income countries, despite the relative ease and remarkable cost effectiveness of proven interventions to prevent this mode of transmission. Progress has been made in sub-Saharan Africa, with an average PMTCT coverage of 58%. However, in some regions of the world, especially in north Africa and in the Middle East, coverage is still sometimes less than 1% [1].

In addition, a small but noteworthy proportion of children are infected due to contaminated blood products and unsafe medical practices, an overlooked tragedy that is entirely preventable. The largest such outbreak was reported in Central China's Henan region and neighbouring provinces in the 1990s, where large-scale blood collection enterprises in these provinces cut corners, reused collection equipment and generated a unique epidemic among adult donors. The children of this iatrogenic epidemic were affected by losing parents, and many perinatally infected infants were born as a secondary effect of this tragedy [2].

More recent HIV outbreaks due to iatrogenic transmission were reported in the central Asian states of Kazakhstan and Uzbekistan in 2006 and 2007. Due to official denial by the government, little is known about the Uzbek outbreak, although it is probably similar to the situation in Kazakhstan. The outbreaks were caused by the use of non-sterile medical equipment and unsafe blood in a corrupt scheme in which parents were persuaded to accept unnecessary blood transfusions for their children. In Kazakhstan, 119 children were confirmed to have been infected with HIV, of whom at least 10 had died by 2007 [3,4].

Millions of children are additionally affected as AIDS erodes the families and communities in which they live. More than 15 million children have lost one or both parents to the disease. Consequently, children suffer the effects of increased poverty, family disruption, interrupted or prematurely terminated education, and additional work, including becoming caregivers. As AIDS continues to affect families, an increasing number of youth-headed households are emerging, with young people assuming the role of breadwinners for their younger siblings [1].

Further, children have to cope with the psychosocial distress caused not only by the presence of serious illnesses affecting family members, but also by discrimination and social exclusion that often accompanies HIV and AIDS [5]. Existing community support mechanisms are eroded by stigmatization of HIV and AIDS and chronic dependency. Exhaustion of financial, social and emotional resources ultimately drives families into poverty and isolation, further exacerbating the health outcomes of family members [6].

Today, there is no question of the urgent need to prevent paediatric HIV and provide treatment to children. However, mere provision of antiretrovirals will not be sufficient; it is of pivotal importance that treatment and care for children are integrated into the broader context of family-support schemes.

The concept of family-centred care and services for children has been increasingly recognized and adopted with regard to other paediatric illnesses, in particular in high-income countries [7]. This philosophy is based on the understanding that a healthy family constitutes the foundation for a child's wellbeing. There is clear evidence showing that children's health outcomes are strongly dependent on those of their parents, caregivers and families. For example, studies show that maternal death and maternal HIV infection increase the risk of child death [8,9].

Despite growing evidence of the benefits of family centred services, reforms in favour of family-oriented HIV interventions have been slow to emerge. The field frequently adopts an individualistic, person-oriented framework, and treatment, prevention and care interventions often target individuals, rather than families and communities [10]. However, we now recognize that infections of individuals ultimately impact the structure of families and society, and that the loss of income of an HIV-infected parent, the burden of healthcare expenses, and the psychosocial stress associated with this disease transcends individuals.

Families, defined in an inclusive way, can and should play a central role in delivery of treatment, prevention and care for children, and family members should be involved in the decision making for any health-related intervention. This approach will be critical to meet the challenges of a growing epidemic, including among the most marginalized groups, many of whom have children.

Investing in programmes that target the entire family will undoubtedly have long-term benefits for our response to HIV. Families are the primary sources of behavioural patterns, and interventions involving the entire family may positively influence risk reduction and health-seeking behaviours, and may help to overcome disparities in access to treatment and healthcare observed between men and women [11,12].

Although progress in expanding access to treatment and support appears moderate, indications of change induce optimism: during recent years, donors have increasingly recognized the need for programmes that specifically target families. PMTCT-plus models have been developed to provide comprehensive care and treatment to HIV-infected, pregnant women and members of their families. Increasing numbers of home-based HIV counselling and testing and treatment programmes are being implemented and gaining ground [13,14].

The international community now needs to reshape its thinking and construct targeted approaches that build on the strengths of families and provide support in a framework for the benefit of the entire family.

The *Journal of the International AIDS Society *is pleased to launch this special issue, which we hope constitutes a beginning of what could be a groundswell of interest in family-centred services for children affected by HIV and AIDS. This is the first time that the rationale for family centred services for children affected by HIV and AIDS and some of the available evidence for its effectiveness has been brought together in one place.

The articles in this issue have been solicited from the initiative, The Road to Vienna, led by the Coalition on Children Affected by AIDS (CCABA). This initiative, which brings together a number of foundations and other partners committed to the wellbeing of children, is striving to accelerate the generation of evidence on the feasibility and effectiveness of family-oriented programmes for children affected by HIV and AIDS, and to promote the implementation of sustainable and effective interventions.

This special issue of nine articles explores the various elements and dimensions of families affected by HIV and AIDS within a variety of contexts.

Beginning with an opening piece by Linda Richter, readers are introduced to the field of family-centred services. Richter presents historical highlights on emergence of this thinking, and provides a definition of the family in the context of the delivery of health services by offering an insight into the complex reality of children affected by HIV and AIDS.

Betancourt *et al *go on to review the evidence for family-centred models for prevention of vertical transmission, exploring the existing evidence and identifying areas for further research.

In a systematic review, Leeper *et al *present an analysis of the impact of family-centred HIV treatment models on children's health outcomes.

Men as fathers, oft-invisible elements of families, are addressed in two papers. Sherr explores the existing literature, covering a broad range of dimensions of HIV in relation to men, their sexuality, their desire for fatherhood and their paternal roles. In their complementary paper, Hosegood and Madhavan closely investigate how men can be successfully included in programmes for women and children in sub-Saharan Africa.

Exemplary cases from Ukraine, Zambia and India are presented in two articles by Beard *et al *and Solomon *et al*. These articles describes the role of families and implications for children of marginalized populations, such as drug users, female sex workers, and married men who have sex with men and women.

HIV interventions for youth, yet another area sufferring from lack of exposure, is the focus of a review by Bhana *et al*. Describing the Collaborative HIV Prevention and Adolescent Mental Health Project, the authors present a model for meeting the needs of pre-adolescents and early adolescents in poverty-affected settings.

Lastly, Tomlinson provides us with a different angle, and examines research from the field of depression to draw lessons for family-centred approaches to children affected by HIV and AIDS.

By publishing this special issue, we hope to make an important contribution to the discourse targeting the broader public including community members, policy makers and academics. Readers have the opportunity to comment on individual articles by scrolling to the end of the article on the website. We would like to invite and encourage readers to contemplate the diverse aspects of this area and to engage with the editors and the authors in dialogue on this important and timely issue.
